# *Comment to:* The Incidence of Fracture-Related Infection in Open Tibia Fracture with Different Time Interval of Initial Debridement

**DOI:** 10.5704/MOJ.2403.020

**Published:** 2024-03

**Authors:** SM Esmat, RY Kow, CL Low

**Affiliations:** 1 Department of Orthopaedics, Hospital Melaka, Melaka, Malaysia; 2 Department of Orthopaedics, Traumatology and Rehabilitation, International Islamic University Malaysia, Kuantan, Malaysia; 3 Department of Radiology, International Islamic University Malaysia, Kuantan, Malaysia

Dear editor,

We read with great interest the study by Hadizie *et al* in the February 2022 issue entitled “The Incidence of Fracture-Related Infection in Open Tibia Fracture with Different Time Interval of Initial Debridement”^1^. In their paper, they presented the relationship between fracture-related infection in open fracture of the tibia with time intervals of initial debridement. With limited studies in Malaysia investigating the correlation between the timing of initial debridement and the incidence of infection in open fracture of tibia, this study potentially shed more light on the optimal timing of initial debridement^2^.

The central tenet in treating open fractures is to prevent infection. Early administration of antibiotic prophylaxis, timely wound debridement, and wound coverage play key roles in preventing infection^3^. Fracture-related infection is a convoluted problem that may be contributed by a multitude of factors such as host immunosuppression, the severity of soft-tissue injury, the experience of surgeon, the presence of other injuries or neurovascular impairment, and post-operative wound care. Although it is widely accepted that the initial debridement of open fracture should be performed early, there was no consensus on the ideal timing of debridement post-trauma.

As discussed in their article, the 6-hour rule of optimal debridement timing stems from initial reports in the 1980s whereby they noticed that the risk of infection was two-fold in patients who were treated 6 hours after the trauma^1^. The results were further strengthened by histological studies that demonstrated the formation of biofilm at 5 hours of exposure and matured within 10 hours^1^. Since then, it was established that the most significant factor that predisposed patients with open tibia fractures to infection was the timing of initial administration of broad-spectrum antibiotics (less than 3-hours after trauma). Previous study by Yusof *et al* in Malaysia showed that a delayed initial debridement would not lead to a higher infection rate, but the fractures were more likely to end up with non-union^2^.

We applaud the effort of authors in collecting such a comprehensive data in their study. Nevertheless, the article falls short of answering the key question due to their data analysis and/or presentation method. In this article, the authors group the timing of initial debridement into categorical data with less than 12 hours as the reference (due to no infected case in the 6-hour group). Do the authors explore the statistical significance between the infection rates in those who have surgeries after 24 hours (9.1%) and those who have their surgeries in less than 24 hours (8.6%)? Alternatively, authors can mimic the analysis by Yusof *et al* whereby they investigate the timing of initial surgery using continuous data instead of categorical data^2^. Furthermore, authors do not break down the severity of the open fracture in each of the timing category, rendering the data invalid owing to the fact that high-grade open fractures are more prone to infection and surgical interventions tend to be performed earlier than those with low-grade open fractures. Among those who had their surgeries done in less than 6 hours, two-third of patients sustained grade IIIa open fractures, whereas less than half of the grade IIIa open fractures were treated after more than 24 hours.

Despite these limitations, this article highlights an important point which is urgent debridement, especially at night with limited supporting staff, is not warranted and the initial debridement can be slightly delayed as the incidence of infection is the lowest when the patient is treated between 6 to 12 hours.
