# Supplementary material for: Comment to: The Incidence of Fracture-Related Infection in Open Tibia Fracture with Different Time Interval of Initial Debridement
Source: Malays Orthop J. 2024 Mar;18(1):153–4. doi: 10.5704/MOJ.2403.020 (PMC11023344; doi:10.5704/MOJ.2403.020)
Supplement: Related texts

# Author’s Reply to the Letter to the Editor

**Published:** 2024-03

**Authors:** Mohd Hadizie Din

**Affiliations:** Corresponding author Department of Orthopaedics, School of Medical Sciences, Universiti Sains Malaysia, Kubang Kerian, 16150, Kota Bharu, Kelantan, Malaysia

Thank you for the constructive comments and suggestions. The aim of our study was to compare the infection rate in open tibial fracture (grade I to IIIa) at different time intervals of initial debridement. In view of that, we were unable to perform continuous data collection and analysis as per your suggestion. Our aim was different than the study by Yusof *et al*.

Apart from that, we did not directly explore the statistical significance between the infection rate in those who had initial debridement after or less than 24-hours. However, we did explore the association between time interval of the initial debridement and infection rate in those who had surgeries less than 12-hours, 12-24-hours and more than 24-hours by using Binary regression analysis. The test cross-comparing between groups and presented as odds of infection (probability of the patient in respective group to have infection). However, the test was not statistically significant.

Lastly, we unable to breakdown the severity of the open fracture in each of the timing category because it will be a very complicated analysis due to small number of samples after dividing them and it will lead to an invalid or wrong statistical analysis. However, the more important facts were to know how many from each category developed infection. And, for those who had complicated by infection, we had discussed it in detail in our discussion.

We do agree that this study has a few limitations as stated in our article. With a larger number of samples, a more valid and precise statistical analysis can be performed.
